# Impact of High Risk for Obstructive Sleep Apnea on Survival after Acute Coronary Syndrome: Insights from the ERICO Registry

**DOI:** 10.5935/abc.20160195

**Published:** 2017-01

**Authors:** Flavia C Maia, Alessandra C. Goulart, Luciano F. Drager, Henrique L. Staniak, Itamar de Souza Santos, Paulo Andrade Lotufo, Isabela M. Bensenor

**Affiliations:** 1 Hospital Universitário - Universidade de São Paulo, São Paulo, SP - Brazil; 2 Instituto do Coração (InCor) - HCFMUSP, São Paulo, SP - Brazil

**Keywords:** Acute Coronary Syndrome, Prognosis, Myocardial Infarction, Survivorship (Public Health), Risk Factors, Sleep Apnea, Obstructive

## Abstract

**Background:**

Obstructive sleep apnea (OSA) is a very often clinical condition that can be associated with high mortality risk, particularly in coronary heart disease (CHD). The diagnosis of OSA is not always accessible via the gold-standard method polysomnography.

**Objective:**

To evaluate long-term influence of the high risk for OSA on fatal and non-fatal outcomes after acute coronary syndrome (ACS) in the Acute Coronary Syndrome Registry Strategy (ERICO) Study using the Berlin questionnaire as a surrogate.

**Methods:**

Berlin questionnaire, a screening questionnaire for OSA, was applied in 639 cases of ACS 30 days after the index event. Cox regression proportional-hazards model was used to calculate the hazard ratio (HR) of all-cause, cardiovascular and CHD (myocardial infarction) mortality, as well as, the combined endpoint of fatal or recurrent non-fatal CHD.

**Results:**

The high-risk group for OSA had higher frequencies of previous personal/family history of CHD and diabetes, in addition to a poorer event-free survival, as compared to the low-risk group (p-log-rank=0.03). The HR for fatal or recurrent non-fatal CHD was 4.26 (95% confidence interval, 1.18 - 15.36) in patients at high risk for OSA compared to those at low risk for OSA after a 2.6-year mean follow-up.

**Conclusions:**

Using Berlin questionnaire, we were able to identify high risk for OSA as an independent predictor of non-fatal reinfarction or CHD mortality in post-ACS individuals in a long-term follow-up.

## Introduction

In addition to the classical cardiovascular risk factors, new risk factors associated with cardiovascular disease (CVD) have been detected in recent years. A promising candidate is obstructive sleep apnea (OSA), a common clinical condition characterized by partial or complete upper airway obstruction during sleep. These obstructive events elicit a series of mechanical, hemodynamic, chemical, neural, and inflammatory responses, with adverse consequences for the cardiovascular system. A recent meta-analysis of prospective cohort studies suggests that severe OSA significantly increases the risk of coronary heart disease (CHD), stroke, and all-cause mortality.^1^ Moreover, subclinical atherosclerosis has been associated with OSA in many reports.^2-5^ OSA may also affect the prognosis of patients with CHD. Some previous studies have shown an association of OSA with a poor long-term prognosis after percutaneous coronary intervention^6,7^ and ST-elevation myocardial infarction (STEMI).^8^ This was not confirmed in another study that evaluated acute coronary syndrome (ACS) in a short follow-up of 6 months.^9^

Polysomnography is the gold-standard test for diagnosis of OSA.^10^ However, its use in large epidemiological studies is limited by its high cost. As a surrogate for polysomnography, several authors have attempted to develop screening questionnaires to detect individuals at high risk for OSA. The Berlin questionnaire, one of the available questionnaires for OSA diagnosis, has been used in other countries and in several studies in Brazil.^11-16^ However, no previous study has evaluated OSA assessed by Berlin questionnaire in a sample of ACS with a long-term follow-up.

We aimed to evaluate the Berlin questionnaire, a screening tool of OSA, as a predictor of long-term survival measured in the Acute Coronary Syndrome Registry Strategy (ERICO Study).

## Methods

### Design and population study

The ERICO Study is an ongoing prospective cohort study that enrolled all consecutive cases of ACS at the hospital affiliated to the University of São Paulo (HU-USP), an academic and teaching hospital situated in the district of Butantan, in the western region of the city. The design and baseline data of the ERICO Study are described in detail elsewhere.^17,18^

Individuals with ACS are treated in the emergency department, the internal medicine ward, or in a general intensive care unit. Patients requiring an interventional procedure are mostly referred to the Instituto do Coração of Hospital das Clínicas. The protocol of the study was approved by the local Institutional Review Board addressing research in human participants. All participants provided written informed consents.

All individuals with suspected ACS were invited to take part in the main study. The clinical interview included questions about education attainment (no formal education, elementary, high-school, or college), marital status (single, married, divorced or widowed), race (White, Mixed Race, Black or Asian), main cardiovascular risk factors, such as self-reported hypertension, diabetes, dyslipidemia, obesity, smoking (never, past or current), family and personal history of CHD, and physical inactivity. Further details about ACS definition are elsewhere.^17,18^

Additional data were obtained on cardiovascular risk stratification, such as urgent or scheduled percutaneous transluminal coronary angioplasty (PTCA) and/or coronary artery bypass graft surgery (CABG), echocardiogram findings and information about medications taken. Three physicians were responsible for reviewing all the medical charts, asking the participants for necessary information on hospital admission, and requesting electrocardiograms, laboratory tests (troponin I, MB-creatine kinase, serum glucose, total cholesterol, HDL/LDL-cholesterol, triglycerides and total blood cell count), and for the in-hospital medical treatment.

Six months and each year after the index event, all participants were contacted by telephone to update the information about their vital status, cardiovascular history, use of medications, depressive symptoms, and physical activity.

### OSA definition

Berlin questionnaire was applied to all participants by trained interviewers, 30 days, 180 days, and one-year after ACS. The Berlin questionnaire includes 10 items divided into categories I (5 questions), II (3 questions) and III (2 questions). Two positive answers to questions 1 to 5 define category I as positive, and 2 positive answers to questions 6 to 8 define category II as positive. Category III is fulfilled if the subject presents hypertension or a body-mass index (BMI) ≥ 30 kg/m^2^. The subject will be classified as having a high risk for OSA if at least two categories are positive.^19^ The sensitivity and the specificity of the Berlin questionnaire for CHD were 70% and 48%, respectively.^19^ Some studies in Brazil have similar results.^11,16^

### Outcomes

We analyzed data from mortality [fatal endpoints: due to all causes, CVD and CHD] and a combined endpoint (fatal or recurrent non-fatal CHD). Each identified event was adjudicated using predefined international criteria.^20,21^ Participants were defined as having death from a cardiovascular cause (CVD mortality) if we identified a cause of death classified in the 10th version of the International Classification of Diseases (ICD-10) chapter IX "Diseases of the circulatory system" or if we identified a cause of death classified as ICD-10 code R57.0 "Cardiogenic shock".^22^

Vital status was investigated periodically by a hot-pursuit strategy during the follow-up. Mortality information was confirmed by official death certificates with the collaboration of the municipal and State's health offices (*Programa de Aprimoramento das Informações de Mortalidade no Município de São Paulo, PRO-AIM,* and *Fundação Sistema Estadual de Análise de Dados-SEADE*, respectively).

### Statistical analysis

Baseline characteristics were analyzed according to OSA risk (low and high). Categorical variables were expressed as proportions (%) and compared using the Chi-square or Fisher's exact tests, as indicated. We tested the probability of distribution of continuous variables by the normality test of Kolmogorov-Smirnov. Once continuous variables were parametric, all were expressed in mean (± standard deviation) and compared by OSA risk groups using Student *t*-test. We performed survival analyses (mean follow-up 2.6 years), considering the following endpoints: fatal (all-cause mortality, CVD mortality, CHD mortality), combined endpoint (recurrent non-fatal or fatal CHD) using Kaplan-Meier analysis with the log-rank test. Cox proportional hazard models for fatal and non-fatal outcomes were built and presented as crude, age-sex adjusted and after multivariate adjustment for age, sex, family history of CHD, previous history of ACS, diabetes (yes or no), dyslipidemia (yes or no), smoking (never, past or current), sedentary lifestyle (yes or no), type of ACS (angina, NSTEMI and STEMI myocardial infarction) and ejection fraction (%) on admission. We did not adjust for the presence of hypertension and obesity because the Berlin questionnaire includes these two risk factors as part of the classification criteria. All tests were two-sided, and p<0.05 was considered significant. The statistical analysis was carried out using the SPSS software, version 22.0.

## Results

**Figure 1:**
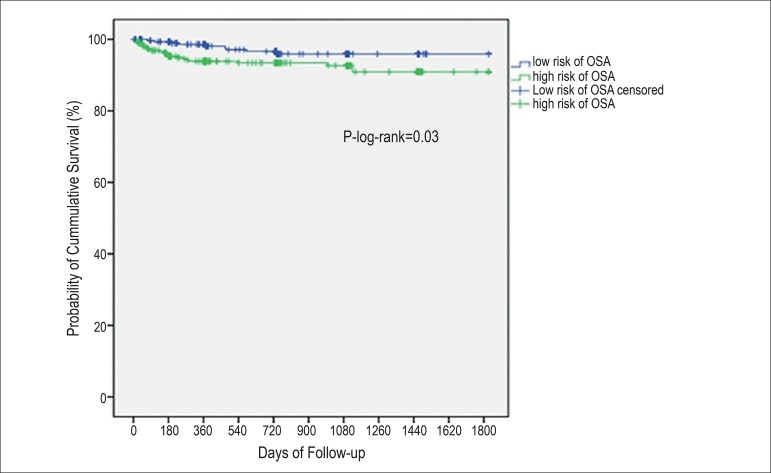
Obstructive sleep apnea as predictor of long-term survival measured by Berlin questionnaire in the ERICO study participants during a 2.6-year mean follow-up.

We included in the present analysis 639 (95.9%) participants with complete information on Berlin questionnaire 30 days after the index event. Individuals detected as having a high risk for OSA according to the Berlin questionnaire were mostly men (55.9%) compared to women (44.1%), p=0.02. In addition, individuals at high risk had higher BMI levels as compared to those at low risk (28.0 versus 25.9 kg/m^2^, p<0.001). We detected higher frequencies of previous history of CHD, obesity, hypertension, diabetes, and sedentary lifestyle in individuals at higher risk for OSA as compared to individuals at low risk. The Kaplan-Meier survival curves were not statistically different comparing individuals classified as at high and low risk for OSA regarding all-cause and CVD mortality or fatal CHD outcomes. However, when we analyzed the composite endpoint (recurrent non-fatal or fatal CHD), the high-risk group for OSA had lower event-free survival than the low-risk group after a mean follow-up of 2.6 years (p-log rank=0.03) (Figure 1). Cox regression analyses confirmed these findings (Table 1).

**Table 1:** General characteristics of the ERICO study participants according to the presence of low- and high-risk for obstructive sleep apnea (OSA) 30 days after acute coronary syndrome

Characteristics	Risk for OSA		p value
	Low n = 310	High n = 329	
Men (%)	201 (64.8)	184 (55.9)	0.02
Mean age (years) (± SD)	62.1 (13.1)	63.1 (12.2)	0.31
Body mass index (kg/m2) (± SD)	25.9 (4.2)	28.0 (5.1)	<0.0001
**Marital status (%)**			0.50
Single	44 (14.2)	35 (10.7)	
Married	189 (61.2)	210 (64)	
Divorced	26 (8.4)	24 (7.3)	
Widowed	50 (16.2)	59 (18)	
**Education (%)**			0.23
No formal education	35 (11.3)	42 (12.8)	
Elementary	183 (59)	198 (60.2)	
High-school	56 (18.1)	66 (20.1)	
College	36 (11.6)	23 (7.0)	
Previous history of coronary heart disease (%)	61 (20.5)	101 (31.9)	0.001
Family history of coronary heart dis-ease (%)	71 (29.2)	102 (39.2)	0.02
Obesity (%)	41 (13.4)	113 (34.8)	<0.0001
Hypertension (%)	182 (59.9)	300 (92)	<0.0001
Diabetes (%)	100 (32.9)	131 (40.7)	0.04
Dyslipidemia (%)	135 (48.7)	168 (56)	0.08
**Smoking (%)**			0.29
Current	102 (33.2)	89 (27.5)	
Past	119 (38.8)	135 (41.7)	
Never	86 (28)	100 (30.9)	
Sedentary lifestyle (%)	201 (67.2)	240 (75.5)	0.02
**Type of acute coronary syndrome (%)**			<0.0001
Angina	74 (23.9)	112 (34.0)	
Non-ST myocardial infarction	127 (41.0)	148 (45.0)	
ST myocardial infarction	109 (35.2)	69 (21.)	
Mean ejection fraction (%) (± SD)	55.8 (13.1)	56.2 (13.2)	0.79

p-values were derived from Chi-Square for categorical variables or Student t-test for continuous variables. SD: standard deviation.

Multivariate adjusted hazard ratios (HR) for high-risk group compared to low-risk group of OSA were calculated for all-cause mortality [HR,1.29; 95% confidence interval (95% CI), 0.64-2.61]; CVD mortality (HR, 1.65; 95% CI, 0.63-4.38), CHD mortality (HR, 2.85; 95% CI, 0.54-15.12) and the composite endpoint (HR, 4.26; 95% CI, 1.18-15.36) (Table 2).

**Table 2:** Hazard ratio and 95% confidence interval of all-cause, CVD and CHD mortality, and combined endpoint including fatal and nonfatal CHD in the ERICO study participants at low and high risk for obstructive sleep apnea

	Crude	Age- and sex- adjusted	Multivariate adjusted
**All-cause mortality**			
Low risk for obstructive sleep apnea	1.0 (Reference)	1.0 (Reference)	1.0 (Reference)
High risk for obstructive sleep apnea	1.17 (0.632.17)	1.31 (0.83-2.07)	1.29 (0.64-2.61)
CVD* mortality			
Low risk for obstructive sleep apnea	1.0 (Reference)	1.0 (Reference)	1.0 (Reference)
High risk for obstructive sleep apnea	1.21 (0.453.24)	1.23 (0.66-2.29)	1.65 (0.63-4.38)
**CHD† mortality**			
Low risk for obstructive sleep apnea	1.0 (Reference)	1.0 (Reference)	1.0 (Reference)
High risk for obstructive sleep apnea	1.21 (0.453.24)	1.24 (0.46-3.34)	2.85 (0.54-15.12)
**Combined endpoint (fatal and recurrent nonfatal CHD)**			
Low risk for obstructive sleep apnea	1.0 (Reference)	1.0 (Reference)	1.0 (Reference)
High risk for obstructive sleep apnea	2.31 (1.06-5.02)	2.34 (1.07-5.08)	4.26 (1.18-15.36)

* CVD: cardiovascular disease;

† CHD: coronary heart disease. Multivariate analysis adjusted for age, sex, diabetes, dyslipidemia, smoking, sedentary lifestyle, previous CHD, family history of CHD, acute coronary syndrome subtype and ejection fraction.

## Discussion

Using the Berlin questionnaire as a surrogate, our results showed a positive association of high-risk for OSA with the composite endpoint including recurrent non-fatal or fatal CHD in patients with a mean follow-up of 2.6 years. The HR of death due to CHD or reinfarction was four times higher among individuals at high-risk as compared to low-risk for OSA. High-risk for OSA measured by Berlin questionnaire was not significantly associated with all-cause mortality, CVD mortality and death due to CHD. A recent meta-analysis by Wang et al.,^1^ including 12 prospective cohort studies in which OSA was diagnosed by polysomnography, showed an association of severe OSA with significantly increased cardiovascular risk, stroke and all-cause mortality. Most of the studies that evaluate OSA as a prognostic factor for cardiovascular events studied specific subsamples of acute and chronic coronary syndrome,^5,9^ STEMI,^8^ unstable angina or CABG,^6,7,23^ and used polysomnography to measure OSA with positive results. However, other studies using simple questions^24^ or specific questionnaires to measure OSA^12,14^ have also found positive associations.^25^ Although some studies in Brazil used the Berlin questionnaire to evaluate the relationship between OSA and other endpoints,^11-13^ only two evaluated the association of OSA defined by the Berlin questionnaire with cardiovascular events.^14,15^ In a prospective cohort study of 200 individuals with ACS, Jesus et al.^14^ evaluated the association of OSA with cardiovascular events using a composite endpoint of cardiovascular death, recurrent CHD events, acute pulmonary edema or stroke. In the multivariate logistic model, a positive association between high risk for OSA and the composite endpoint was reported (OR, 3.66; 95% CI, 1.22-11.0).^14^ Our study has several similarities with that by Jesus et al. Both studies showed a positive association of OSA in a sample of ACS patients using composite endpoints of mortality and morbidity - though not exactly the same - and the same strategy for multivariate adjustment. However, one very important point is that in the study by Jesus et al.,^14^ follow-up was restricted to the period of hospitalization in contrast to the 2.6-year mean follow-up of our analysis. In addition, there are differences in the structure of the two hospitals where the studies were conducted. The study performed by Jesus et al.^14^ was conducted in a reference hospital with a proper hemodynamic facility, while ours was conducted in a general hospital that provides healthcare for people living in the Butantan borough, using the Instituto do Coração (INCor) as the reference center for cardiology. More recently, Correia et al.^15^ tested the hypothesis that clinical suspicion of OSA is an independent predictor of worse in-hospital outcomes in patients with non-ST-elevation ACSs. Presence of a high risk for OSA was positively associated with risk for a cardiovascular event (OR, 3.4; 95% CI, 1.3-9.0), but the follow-up was also restricted to the period of hospitalization.^15^ Our results have also found that OSA is associated with a poorer prognosis in ACS. However, we found that this association exists, including all types of ACS. We were probably underpowered to evaluate prognosis according to the ACS subtype, but as follow-up of the study continues, we may address this objective in a future analysis.

The ERICO study has some diverse characteristics compared to other studies that evaluate ACS worldwide. The HU-USP is a general community-based hospital that cares for the community living in the Butantan borough. Even in that setting, we showed a positive association with the composite endpoint after a 2.6-year mean follow-up after the index event. However, we also have some important limitations that should be noted. The Berlin questionnaire performs more poorly than polysomnography in patients with CHD.^25^ However, full polysomnography is a costly tool, and not readily available at all facilities. This important obstacle, together with the lack of an efficient and easy tool for OSA screening, may partially explain the under-diagnosis of OSA in the cardiology setting.^26^ In our study, the Berlin questionnaire was applied 30 days after the ACS. Therefore, there is probably a survival bias in this analysis, with patients with more severe ACS, and probably also with a higher frequency of OSA, dying before they could enter the study. Even in this circumstance, we found a positive association that suggests real causality between high-risk OSA and CHD combined endpoint. This study also reports some interesting new data, as there have been few studies that exclusively evaluate the relationship of severe OSA with cardiovascular events only for ACS patients with a long-term follow-up. Another important point is the strict criteria used to define ACS, and the statistical analysis that used Cox proportional hazards, which are positive points of this analysis.

## Conclusions

This prospective cohort of CHD demonstrates that a high risk for OSA, measured by the Berlin questionnaire, was an independent predictor of reinfarction or CHD mortality among individuals with ACS after a 2.6-year follow-up.
